# The association of glycogen synthase kinase-3beta (*GSK-3β)* gene polymorphism with kidney function in long-term lithium-treated bipolar patients

**DOI:** 10.1186/2194-7511-1-8

**Published:** 2013-06-20

**Authors:** Janusz K Rybakowski, Maria Abramowicz, Aleksandra Szczepankiewicz, Michal Michalak, Joanna Hauser, Stanislaw Czekalski

**Affiliations:** Department of Adult Psychiatry, Poznan University of Medical Sciences, ul. Szpitalna 27/33, Poznan, 60-572 Poland; Psychiatric Genetics Unit, Poznan University of Medical Sciences, ul. Szpitalna 27/33, Poznan, 60-572 Poland; Laboratory of Molecular and Cell Biology, Poznan University of Medical Sciences, ul. Szpitalna 27/33, Poznan, 60-572 Poland; Department of Computer Science and Statistics, Poznan University of Medical Sciences, ul. Dabrowskiego 79, Poznan, 60-529 Poland; Department of Nephrology, Transplantology and Internal Diseases, Poznan University of Medical Sciences, ul. Przybyszewskiego 49, Poznan, 60-355 Poland

**Keywords:** Glycogen synthase kinase-3beta, -50 C/T polymorphism, Lithium, Kidney function, Urine concentrating ability

## Abstract

**Background:**

Most bipolar patients experience a reduction in urinary concentrating ability within a few weeks of starting lithium treatment. This phenomenon may be connected with the effect of lithium on the glycogen synthase kinase-3beta (GSK-3β) present in the renal tubules. The *GSK-3β* gene is located on chromosome 3q13 and possesses a functional -50 C/T polymorphism. In the present study, we estimated this polymorphism in a group of long-term lithium-treated patients and assessed its association with various parameters of kidney function, including novel markers of kidney injury such as serum neutrophil gelatinase-associated lipocalin (NGAL) and urinary beta2-microglobulin (β2-MG).

**Methods:**

The study comprised 78 patients with bipolar mood disorder (25 males, 53 females), aged 36 to 82 (60 ± 11) years. The mean duration of bipolar illness was 6 to 50 (24 ± 10) years, and the patients have been receiving lithium for 5 to 38 (16 ± 9) years. All the patients had the following features, regarded as the phenotypes of kidney functions measured: urine examination for specific gravity evaluation, serum creatinine concentration, and estimated glomerular filtration rate (eGFR) evaluation, as well as the serum concentrations of NGAL and urinary β2-MG. Genotyping of *GSK-3β* gene -50 C/T polymorphism was done by polymerase chain reaction analysis.

**Results and discussion:**

Thirty-four patients (6 males, 28 females) had the T/T genotype, 37 patients (16 males, 21 females) had the T/C genotype, and 7 patients (3 males, 4 females) had the C/C genotype. Patients homozygous for C allele had significantly higher urine specific gravities (1.019 ± 0.008) compared to the remaining genotypes (1.013 ± 0.007) (*p* = 0.035), with no influence of the duration of lithium treatment. Other parameters of kidney function (serum creatinine, eGFR, serum NGAL, and urinary β2-MG levels) were not different between genotypes and, again, were not affected by the duration of lithium treatment. There was no correlation between urine specific gravity and other kidney function parameters. The results of our study indicate that the *GSK-3β* genotype may be connected with lithium-induced impairment of renal concentrating ability in long-term lithium-treated bipolar patients. Limitations of the study include small size of the sample, small number of C/C genotype patients, and a lack of multiple testing analysis of genotypic differences in various measures of kidney function.

## Background

Lithium is the most effective therapeutic modality for the prevention of manic and depressive recurrences in bipolar disorder (Rybakowski 2011). Since its introduction for this purpose (Hartigan 1963), considerable concerns have been aroused about a possible negative effect of lithium on kidney function. Evidence of this effect has accumulated from patients receiving lithium for 10 years or more.

The most frequent renal lithium effect is a decrease in urinary concentrating ability, which clinically manifests itself by polyuria and polydipsia of various intensities. It may affect 30% to 80% of patients on long-term lithium therapy and may occur as early as after several weeks of lithium administration (Schou and Vestergaard 1988). In some patients receiving lithium for 10 to 20 years, chronic interstitial nephropathy may develop, which results in increased serum creatinine and a reduction in the glomerular filtration rate (GFR). The duration of lithium treatment is the main predisposing factor for nephropathy which, in a small proportion of patients, can result in end-stage renal disease (Presne et al. 2003). In recent years, some novel markers of kidney injury have been identified. Among these, the serum level of the neutrophil gelatinase-associated lipocalin (NGAL) may be a biomarker for glomerular filtration and correlates with the severity of the damage in patients with chronic kidney disease. Another marker is beta2-microglobulin (β2-MG), the concentration of which in the urine may be correlated with the degree of tubular reabsorption impairment (Lisowska-Myjak 2010; Adiyanti and Loho 2012).

Glycogen synthase kinase-3beta (GSK-3β) is a pluripotent kinase involved in a number of cell functions. The inhibition of GSK-3β by lithium has been discovered nearly two decades ago (Stambolic et al. 1996). From a bipolar disorder perspective, the most important function of GSK-3β may be its role in synaptic plasticity, apoptosis, and the circadian cycle (Bradley et al. 2012; Iitaka et al. 2005). Therefore, the effect on GSK-3β could be responsible for lithium's therapeutic action in bipolar illness, including neuroprotection and circadian regulation (Gould and Manji 2005). In the renal tubules, GSK-3β controls water transport via aquaporin 2 (AQ2) and sodium transport via the epithelial sodium channel. Lithium, unlike sodium, is not transported out of the cells of collecting ducts by the Na+, K+, ATPase and may accumulate intracellularly, reaching a concentration capable of substantially inhibiting GSK-3β. It has been hypothesized that such a mechanism may underlie the lithium-induced impairment of urinary concentrating ability (Grünfeld and Rossier 2009).

The *GSK-3β* gene is located on chromosome 3q13 and possesses a functional -50 C/T promoter polymorphism (rs334558), where a wild T allele has significantly higher transcriptional activity than its mutant C allele (Kwok et al. 2005). In most studies on the association of this polymorphism with the effect of lithium treatment, a better effect has been demonstrated for carriers of C allele of this polymorphism. Such a connection was found in two studies on lithium prophylactic efficacy (Benedetti et al. 2005; Lin et al. 2013). However, in our paper (Szczepankiewicz et al. 2006), we were not able to confirm this. Adli et al. (2007) reported that carriers of the C allele showed a significantly better response to lithium augmentation of antidepressants compared to patients with the T/T genotype. Recently, Italian investigators have showed an association of the C allele of this polymorphism with a lithium-induced increase of white matter in bipolar patients (Benedetti et al. 2013).

We hypothesized that this functional polymorphism of the *GSK-3β* gene could also be associated with the degree of lithium effect on kidney function, including urinary concentrating ability and also, perhaps, other effects. Therefore, in this present study, several parameters of kidney function in a group of long-term lithium-treated patients were assessed in relation to their GSK-3β genotype.

## Methods

### Patients

The study comprised 78 patients with bipolar mood disorder (25 males, 53 females), aged 36 to 82 (60 ± 11) years. The mean duration of bipolar illness was 6 to 50 (24 ± 10) years. Consensus diagnosis by at least two psychiatrists was made for each patient, according to DSM-IV criteria (SCID) (First et al. 1996). These patients have been receiving lithium for 5 to 38 (16 ± 9) years. The study was approved by the Bioethics Committee, Poznan University of Medical Sciences, and all the patients gave their informed consent, after the nature of the procedures had been fully explained to them.

### Measurements of kidney function

The following investigations, regarded for the use of this study as the phenotypes of kidney function were carried out: urine examination with specific gravity evaluation performed in a sample collected after overnight hydropenia, serum creatinine concentration, and estimated GFR (eGFR) evaluation, as well as two markers of kidney injury: the serum concentration of NGAL and the urinary concentration of β2-MG.

The serum concentration of creatinine was measured by the enzymatic method with creatininase. The eGFR was calculated according to the MDRD formula (Kokot et al. 2011). The NGAL in serum was measured by the enzyme-linked immunosorbent assay method, and the β2-MG in urine was assessed by the enzymatic immunoassay method.

### Genotyping

The DNA was extracted from 10 ml of EDTA-anticoagulated whole blood, using the salting out method (Miller 1988). A 344-bp fragment of the *GSK-3β* gene was amplified by polymerase chain reaction (PCR) reaction, with the set of primers described by Russ (2001), using a PTC-200 (MJ Research, St. Bruno, Canada) thermal cycler. The PCR product (4.0 ml) was then digested with *Alu*I restriction endonuclease (MBI Fermentas, Burlington, Canada). The uncut PCR product size was 344 bp. After RFLP analysis, the following alleles were observed: uncut C allele (127 bp), and for the T allele, the bands of 220 and 124 bp. To confirm the results, the restriction analysis for the uncut C allele was repeated.

### Statistics

The results are presented as the means ± SD for the whole group of patients and for the two subgroups of patients: men and women. Comparisons of the subgroups were performed by the one-way analysis of variance (ANOVA) test and by the unpaired *t* test for the two groups. The distribution of genotypes according to the Hardy-Weinberg equilibrium was determined for the whole group and, additionally, for each gender. The chi-square test was used for calculating differences in genotypic distribution. Any possible association of genotypes of the *GSK-3β* gene with parameters of kidney function was analyzed, also taking into account the duration of lithium treatment, as a covariant (analysis of covariance). For correlations, Pearson's linear correlation coefficient was used. The significance of the correlation coefficient was checked by Student's *t* test. All the calculations were made using the Statistica (StatSoft, Polska) version 10 statistical package. The level of statistical significance was determined at *p* < 0.05.

## Results

The measurements of kidney function in the whole group of patients studied, as well as in the male and female subgroups, are shown in Table 1.

**Table 1:** **Measurements of kidney function in the patients studied**

	Total	Men	Women
	***N*** = 78	***N*** = 25	***N*** = 53
Urine specific gravity	1,013 ± 0.007	1,013 ± 0.008	1,013 ± 0.008
Serum creatinine (mg/dl)	0.95 ± 0.25	1.18 ± 0.28	0.83* ± 0.17
Estimated glomerular filtration rate (ml/min/1.73 m^2^)	71 ± 14	67 ± 18	73 ± 12
Serum neutrophil gelatinase-associated lipocalin (ng/ml)	300 ± 118	326 ± 112	287 ± 119
Urine beta2-microglobulin (μg/ml)	0.18 ± 0.19	0.20 ± 0.20	0.17 ± 0.18

Values are expressed as means ± SD. *p < 0.001, significant difference between male and female patients (*t* test).

A significantly higher serum creatinine concentration was found in the male patients than in females (*p* < 0.001). This was reflected in a lower eGFR in the male patients, at the level of statistical trend (*p* = 0.085). Correlation with the duration of lithium therapy was obtained only in the group of male patients: a positive correlation with beta2-microglobulin (*p* = 0.005) and creatinine concentration (*p* = 0.048) and a negative correlation with urinary specific gravity (*p* = 0.030).

The genotypic distributions of -50 C/T polymorphism of the *GSK-3β* gene in the patients studied, with their Hardy-Weinberg equilibrium values, are shown in Table 2.

**Table 2:** **Genotype distribution in the group of patients studied with HWE - frequencies and** ***p*** **values**

	T/T	T/C	C/C	HWE
Total (*N* = 78)	34 (44%)	37 (47%)	7 (9%)	0.491
Male (*N* = 25)	6 (24%)	16 (64%)	3 (12%)	0.135
Female (*N* = 53)	28 (53%)	21* (39.5%)	4 (7.5%)	0.981

*p = 0.025, significant difference between male and female patients as to T/T vs. T/C distribution (chi-square test). HWE, Hardy-Weinberg equilibrium.

Thirty-four patients (6 males, 28 females) had the T/T genotype, 37 patients (16 males, 21 females) the T/C genotype, and 7 patients (3 males, 4 females) the C/C genotype. The genotypes were in Hardy-Weinberg equilibrium, both for the whole group as well as for the male and female subgroups. The T/T genotype was significantly less frequent in males, while the T/C genotype was significantly less frequent in the females (*χ*^2^ = 5.05, *p* = 0.025).

The analysis of the genotypic differences, in relation to kidney function, is shown in Table 3. Due to significant differences in serum creatinine concentration, the analysis of this parameter was additionally performed separately for the male and female subjects.

**Table 3:** **Analysis of genotype differences in relation to kidney function**

	T/T	T/C	C/C	T/T vs. T/C vs. C/C	T/T vs. T/C + C/C	T/T + T/C vs. C/C
	***N*** = 34	***N*** = 37	***N*** = 7	(ANOVA)	***t*** test	***t*** test
Urine specific gravity	1,012 ± 0.007	1,013 ± 0.007	1,019 ± 0.008	*p* = 0.052	*p* = 0.215	*p = 0.035*
Serum creatinine, total (mg/dl)	0.89 ± 0.20	0.99 ± 0.28	0.98 ± 0.23	*p* = 0.448	*p* = 0.074	*p* = 0.603
Male	1.19 ± 0.25	1.16 ± 0.32	1.22 ± 0.10	*p* = 0.939	*p* = 0.884	*p* = 0.781
	*N* = 6	*N* = 16	*N* = 3			
Female	0.83 ± 0.12	0.85 ± 0.14	0.80 ± 0.06	*p* = 0.774	*p* = 0.772	*p* = 0.515
	*N* = 28	*N* = 21	*N* = 4			
Estimated glomerular filtration rate (ml/min/1.73 m^2^)	71 ± 13	71 ± 16	68 ± 9	*p* = 0.977	*p* = 1.000	*p* = 0.606
Serum neutrophil gelatinase-associated lipocalin (ng/ml)	288 ± 89	321 ± 142	248 ± 81	*p* = 0.441	*p* = 0436	*p* = 0.216
Urine beta2-microglobulin (μg/ml)	0.22 ± 0.24	0.16 ± 0.14	0.13 ± 0.05	*p* = 0.279	*p* = 0.162	*p* = 0.410

The *p* values of the differences in kidney function values are expressed as means ± SD. Italicized *p* value indicates urine specific gravity significantly higher in C/C patients compared to remaining genotypes combined.

Patients homozygous for the C allele had significantly higher urine specific gravity (1.019 ± 0.008) compared to the remaining genotypes (1.013 ± 0.007) (*p* = 0.035), with no influence of the duration of lithium treatment. Other parameters of kidney function (serum creatinine, eGFR, NGAL, and urinary β2-MG levels) were not different between genotypes, irrespective of the duration of lithium treatment. CC homozygotes had numerically lower values of serum NGAL and urine β2-MG compared to other genotypes. However, this difference was not statistically significant.

In this group of patients, there was no correlation between urine specific gravity and other parameters of kidney function.

## Discussion

The main finding of this study is that lithium-treated patients homozygous for the C allele have better preserved function of renal concentrating ability, compared to the remaining genotypes of this polymorphism, as reflected in higher urinary specific gravity in such patients. This may mean that C allele-bearing patients may be more resilient to the lithium inhibitory effect on GSK-3β activity in kidneys.

GSK-3β is an enzyme which has recently gained increasing attention as a signaling molecule in the epithelial cells of renal tubules as its inhibition may have various consequences. On the one hand, it has been shown that the inhibition of such a proapoptotic and antiproliferative signaling factor as GSK-3β could be protective in acute kidney injury (Dugo et al. 2005). On the other hand, the inhibition of this enzyme could lead to a disturbance of water homeostasis in the kidneys, mainly by disturbed AQ2 regulation of water channels via adenylate cyclase or the prostaglandin E2 pathway. In the short term, GSK-3β could disrupt vasopressin signaling in the collecting duct cells and, in the long term, make the collecting duct less responsive to the hydro-osmotic effect of vasopressin (Rao 2012).

As mentioned earlier, other clinical studies in which this *GSK-3β* gene polymorphism was estimated have shown a positive association between possessing the C allele and lithium therapeutic effects such as the quality of long-term prophylaxis of bipolar mood disorder (Benedetti et al. 2005; Lin et al. 2013), augmentation of antidepressants in treatment-resistant depression (Adli et al. 2007), or lithium-induced increase of white matter in bipolar patients (Benedetti et al. 2013). The better effect of lithium in patients with the C allele could be explained by a detrimental effect of GSK-3β on brain function and a greater inhibitory effect of lithium in patients with lower activity of the enzyme. As to kidney function, an effect of the C allele on lithium-induced GSK-3β inhibition is more difficult to explain. In view of the dual effects of GSK-3β inhibition on kidney cells, it may be hypothesized that, in C allele homozygotes, a protective effect of this inhibition prevails, while in other genotypes, such an effect leads mostly to an impairment of the urinary concentrating ability. It seems therefore that the mutant C allele of *GSK-3β* gene polymorphism may not only be connected with a better clinical lithium response due to the brain effect of this drug, but that also it is protective against the lithium effect on some aspect of kidney function.

In this group of patients, we demonstrated a significant relationship of the *GSK-3β* genotype only with one aspect of kidney function, the urinary concentrating ability. We did not find any significant association in the remaining parameters measured. However, some hint could come from the numerically lower values of serum NGAL and urinary β2-MG in the CC homozygotes compared to other genotypes. These values could achieve statistical significance with a higher number of patients possessing the C/C genotype.

The main limitation of our study is the small size of the sample and especially the small number of C/C genotype patients (9%). Furthermore, a multiple testing analysis was not applied to genotypic differences in relation to various measures of kidney function. In such case, after Bonferroni correction, the main result connected with urine specific gravity would be insignificant. However, if treating urine specific gravity as a separate parameter, we believe that the difference between C/C and other phenotypes combined (1.019 ± 0.008 vs. 1.013 ± 0.007, respectively) is a significant finding. On the other hand, the strength of our study is the long duration of lithium administration given to our patients, which allows us to make a true assessment of the long-term effects of lithium on kidney function.

## Conclusions

The results of our study indicate that the *GSK-3β* genotype may be connected with lithium-induced impairment of renal concentrating ability in long-term lithium-treated bipolar patients. This may reflect a putative role of this enzyme in the mechanisms of water and sodium transport in the renal tubules. None of the other aspects of kidney function studied appeared to be influenced by this genotype. However, bearing in mind the limitations of our study, we should regard it as an exploratory one warranting further studies in larger samples.

## Abbreviations

ANOVA: Analysis of variance
AQ2: Aquaporin 2
β2-MG: Beta2-microglobulin
EDTA: Ethylenediaminetetraacetic acid
GFR: Glomerular filtration rate
GSK-3β: Glycogen synthase kinase-3beta
NGAL: Neutrophil gelatinase-associated lipocalin
PCR: Polymerase chain reaction
RFLP: Restriction fragment length polymorphism
SCID: Structured clinical interview for DSM disorders.
